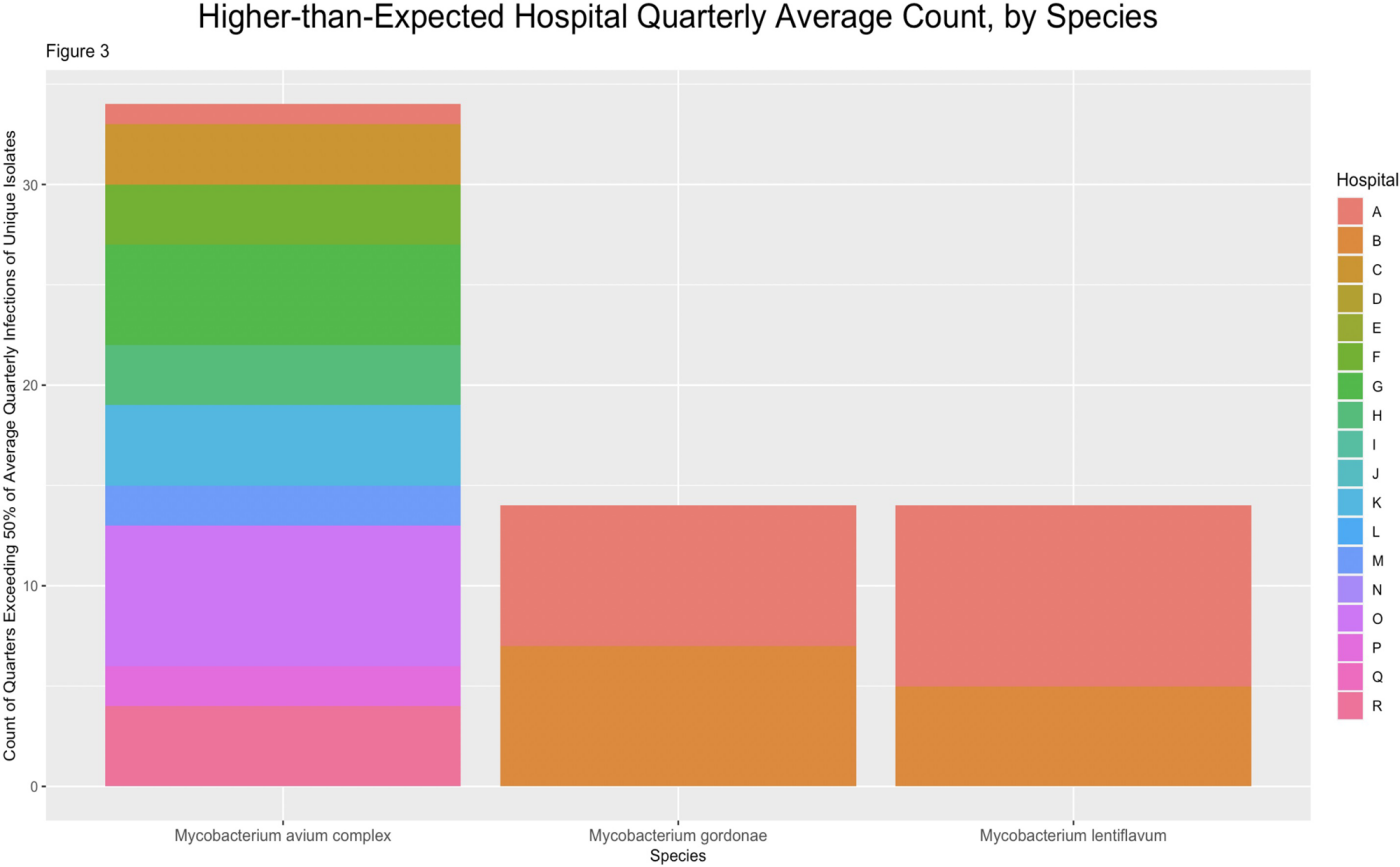# Multi-year Epidemiology of Nontuberculous Mycobacteria Across a Diverse Healthcare System

**DOI:** 10.1017/ash.2024.291

**Published:** 2024-09-16

**Authors:** Spencer Schrank, Lee Harrison, Elise Martin, Graham Snyder

**Affiliations:** UPMC; University of Pittsburgh; VA Pittsburgh Healthcare System

## Abstract

**Background:** Nontuberculous mycobacteria (NTM) are ubiquitous potential pathogens implicated in healthcare-associated outbreaks. There is a paucity of studies describing transmission risk in the healthcare setting. To estimate the potential healthcare-associated transmission of NTM, we characterized the frequency of NTM clinical isolates across our multi-facility healthcare system. **Method:** We performed a retrospective review of all clinical NTM isolates at 21 healthcare facilities in a large health system between January 2019 through June 2023 (inclusion criteria: all first unique species). We analyzed the quarterly frequency of isolates for each species, by facility. We identified higher-than-expected species frequencies, which was defined as a quarterly frequency ≥50% higher than the average quarterly frequency for that facility, for the entire study period (analysis omitted for any hospital with an average quarterly frequency 10 unique patient isolates in any 12-month period or >2 in a single month except for M. abscessus at Hospital A. The quarterly frequency of the three most common species among hospitals with ≥2 unique isolates per 12-month period are displayed in figure 2. An increase of 50% from the average quarterly infection count occurred 34 times for M. avium complex across 10 hospitals, 14 times for M. gordonae across 2 hospitals and 14 times for M. lentiflavum across 2 hospitals (Figure 3). **Conclusion:** A diverse group of NTM were isolated across our healthcare system over the study period, most commonly M. avium complex, M. gordonae, and M. lentiflavum, each with hospital-specific temporal frequencies that suggest the potential for undetected outbreaks, while frequencies of less commonly isolated species were rarely suggestive of potential undetected outbreaks. Further epidemiologic investigation of in-hospital transmission routes, with whole genome sequencing to determine genetic relatedness, is necessary to identify undetected outbreaks.

**Figure:**
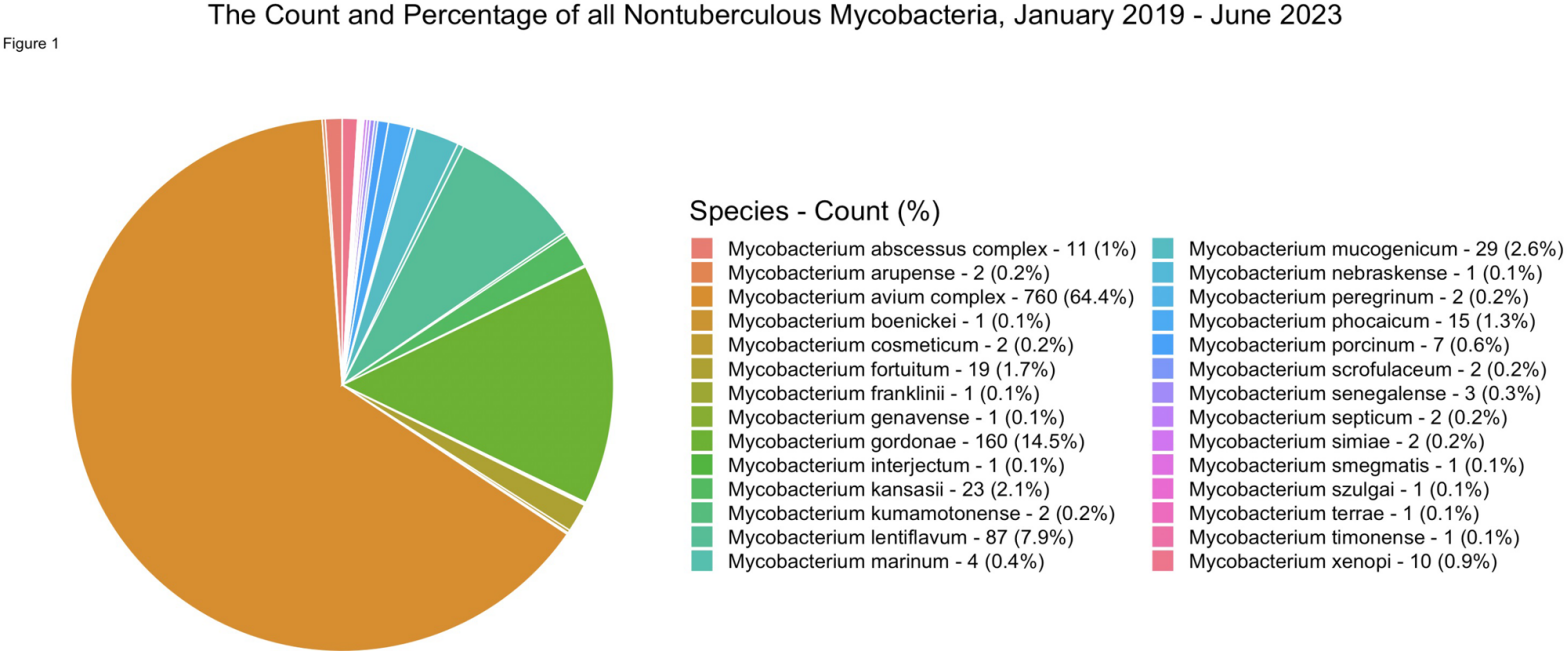

**Figure:**
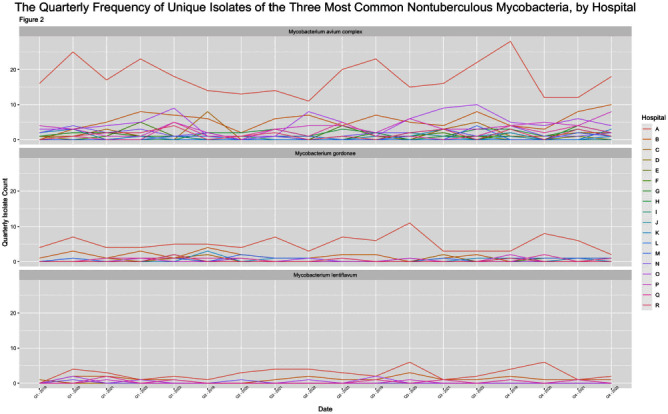

**Figure:**